# Draft Genome Sequence of Multidrug-Resistant Listeria innocua Strain UAM003-1A, Isolated from a Wild Black Bear (Ursus americanus)

**DOI:** 10.1128/MRA.01281-19

**Published:** 2019-11-21

**Authors:** Cameron Parsons, Yi Chen, Jeffrey Niedermeyer, Kevin Hernandez, Sophia Kathariou

**Affiliations:** a Department of Food, Bioprocessing, and Nutrition Sciences, North Carolina State University, Raleigh, North Carolina, USA; b Division of Microbiology, Center for Food Safety and Applied Nutrition, Food and Drug Administration, College Park, Maryland, USA; University of Rochester School of Medicine and Dentistry

## Abstract

There is currently limited knowledge of the genome sequences of nonpathogenic *Listeria* species, especially strains from wildlife. Here, we report the draft genome sequence and associated genome information of an antibiotic-resistant Listeria innocua strain, UAM003-1A, isolated from the feces of a black bear in California, USA.

## ANNOUNCEMENT

The genus *Listeria* is distributed widely in nature (1, 2). Listeria monocytogenes, the only human pathogen in the genus, is a leading cause of death due to foodborne disease (listeriosis) in the United States and other industrialized nations (3, 4), with severe health outcomes including septicemia, meningitis, and stillbirths (5). Listeria innocua and other *Listeria* spp. are nonpathogenic but can serve as important reservoirs for resistance determinants that can transfer to L. monocytogenes (6). They can therefore be of particular concern if there is transmission of antibiotic resistance to a pathogen of major human health concern.

Here, we report the genome sequence and antimicrobial resistance (AMR) of Listeria innocua UAM003-1A, isolated in May 2017 using the previously described ISO method, which involved culturing the feces of a black bear (Ursus americanus) captured in California, first with primary enrichment (24 to 48 h) in Half Fraser broth, followed by transfer to Full Fraser broth for secondary enrichment for 24 to 48 h (7). The lack of hemolysis on blood agar indicated that this strain was likely a *Listeria* species other than *monocytogenes*.

The strain was grown aerobically overnight at 37°C in brain heart infusion broth (Becton, Dickinson and Company, Franklin Lakes, NJ, USA), and genomic DNA was extracted using a DNeasy blood and tissue kit (Qiagen, Valencia, CA); libraries were prepared using 1 ng of genomic DNA with the Nextera XT DNA library preparation kit (Illumina, San Diego, CA, USA), and the genome was sequenced using the NextSeq 500 desktop sequencer with the NextSeq 500/550 high-output v.2 kit (300 cycles) (Illumina) for 2 × 151 cycles according to the manufacturer’s instructions. Sequencing resulted in 3,282,404 150-bp paired-end reads. Raw sequencing reads were quality-trimmed and *de novo*-assembled using Spades v.3.3.13 (8) with an average coverage of 141.406×. Assembly was quality-assessed using QUAST v.4.6.4 (9) and interrogated for AMR genes using the ResFinder database (10) in ABRicate (https://github.com/tseemann/abricate). Default parameters were used for all software.

The genome was assembled into 27 contigs with a total length of 2.89 Mb, an *N*_50_ value of 480,020 bp, and an average GC content of 37.45%. Whole-genome sequence analysis, including *in silico* multilocus sequence typing (MLST) via BIGSdb-*Lm* (https://bigsdb.pasteur.fr/listeria/), confirmed that this strain was *L. innocua* with the novel sequence type 1495 (ST1495) in clonal complex (CC) 140, which also includes the *L. innocua* reference strain CLIP11262 (11). ResFinder analysis revealed three putative antibiotic resistance determinants, *tet*(M), *ant(6)-Ia_2*, and *mphB*, known to confer resistance to tetracycline, aminoglycosides, and macrolides, respectively. Interestingly, each determinant was harbored on a different mobile genetic element, with *tet*(M) and *ant(6)-Ia_2* on separate transposons, while *mphB* appeared to be harbored on a prophage. Testing for antimicrobial resistance was done as previously described (12) against a panel of antibiotics, the heavy metals cadmium and arsenic, and the quaternary ammonium disinfectant benzalkonium chloride. Testing confirmed that *L. innocua* UAM003-1A was resistant to tetracycline (MIC, >5 μg/ml) and streptomycin (MIC, >20 μg/ml) but not other aminoglycosides, such as kanamycin and gentamicin, while it lacked resistance to the macrolide erythromycin (MIC, >0.5 μg/ml). The sequence data will further elucidate AMR emergence and functionality/specificity of AMR genes in *Listeria* spp. The public and animal health relevance of *L. innocua* UAM003-1A is enhanced by current demographic trends that are enhancing the likelihood of human contact with black bears and other wildlife.

### Data availability.

The whole-genome sequence of *L. innocua* strain UAM003-1A was deposited at DDBJ/EMBL/GenBank under the accession number VNKM00000000. The version described in this paper is version VNKM01000000. The raw sequence reads were deposited in the SRA under accession number PRJNA556464.
